# MANet: a multi-attention network for automatic liver tumor segmentation in computed tomography (CT) imaging

**DOI:** 10.1038/s41598-023-46580-4

**Published:** 2023-11-16

**Authors:** Kasun Hettihewa, Thananop Kobchaisawat, Natthaporn Tanpowpong, Thanarat H. Chalidabhongse

**Affiliations:** 1 Perceptual Intelligent Computing Laboratory, Department of Computer Engineering, Faculty of Engineering, Chulalongkorn University, Bangkok, 10330 Thailand; 2 Eikonnex AI Co., Ltd., Bangkok, Thailand; 3 Department of Radiology, Faculty of Medicine, Chulalongkorn University, Bangkok, 10330 Thailand; 4 Applied Digital Technology in Medicine (ATM) Research Group, Faculty of Engineering, Chulalongkorn University, Bangkok, 10330 Thailand

**Keywords:** Computer science, Cancer imaging

## Abstract

Automatic liver tumor segmentation is a paramount important application for liver tumor diagnosis and treatment planning. However, it has become a highly challenging task due to the heterogeneity of the tumor shape and intensity variation. Automatic liver tumor segmentation is capable to establish the diagnostic standard to provide relevant radiological information to all levels of expertise. Recently, deep convolutional neural networks have demonstrated superiority in feature extraction and learning in medical image segmentation. However, multi-layer dense feature stacks make the model quite inconsistent in imitating visual attention and awareness of radiological expertise for tumor recognition and segmentation task. To bridge that visual attention capability, attention mechanisms have developed for better feature selection. In this paper, we propose a novel network named Multi Attention Network (MANet) as a fusion of attention mechanisms to learn highlighting important features while suppressing irrelevant features for the tumor segmentation task. The proposed deep learning network has followed U-Net as the basic architecture. Moreover, residual mechanism is implemented in the encoder. Convolutional block attention module has split into channel attention and spatial attention modules to implement in encoder and decoder of the proposed architecture. The attention mechanism in Attention U-Net is integrated to extract low-level features to combine with high-level ones. The developed deep learning architecture is trained and evaluated on the publicly available MICCAI 2017 Liver Tumor Segmentation dataset and 3DIRCADb dataset under various evaluation metrics. MANet demonstrated promising results compared to state-of-the-art methods with comparatively small parameter overhead.

## Introduction

Liver cancer is one of the major cancer types with the most fatalities recorded around the world^1, 2^. For immediate clinical management to be successful in achieving survival, early detection of liver tumors is essential. Tumor burden analysis which consists of major factors of measuring the size and location of the tumor, utmost importance to determine the severity of the disease. Medical imaging is a noninvasive technique to determine the severity and stratification of cancer. Radiologists mostly rely on Computed Tomography (CT) scans for the diagnosis and clinical management prior to the pathological examination. It is because of the contrast enhancement on CT images that can be helpful to distinguish the tumor region from the liver parenchyma. However, recognizing tumor regions is still a challenging task for radiologists due to high inter-class similarity, intra-class variations, and fussy boundaries of the tumors. To address these issues, computer-aided detection system is highly useful to establish diagnostic standards to bridge the cognition gap in all levels of radiological expertise.

There are some challenges still remaining to develop computer-aided automatic liver tumor segmentation solution. The high cost of collecting data to conduct experiments. Data labeling is time-consuming and tedious task to prepare proper medical dataset to train and test the model. Another major issue that causes the misclassification of tumor regions is tumor diversity. Tumor can appear in different shapes at different locations with different quantities. The intensity dissimilarity in tumor regions increases the complexity to differentiate tumor regions from the healthy liver.

To address those challenges, researchers have attempted to develop segmentation methods with various deep learning techniques. Medical image analysis has significantly developed with convolutional neural networks (CNN), which have noticeably improved performance on a wide range of computer vision tasks by automatically learning multi-level feature representations. The rapid development in deep learning, fully convolutional network (FCN) emerged with remarkable accuracy in pixel-level classification, which is proposed by Long et al.^3^. Fully connected layers of CNN are replaced with convolutional layers to perform the pixel-level classification. Ronneberger et al.^4^ proposed U-Net, this model is based on FCN and could achieve massive success in medical image segmentation. Significant advancements were made by researchers who inspired by U-Net. H-DenseUnet proposed by Li et al.^5^ is a recent development of U-Net as a combination of U-Net with DenseNet^6^ for efficiently extracting intra-slice and inter-slice features. Zhou et al.^7^ proposed U-Net++ based on nested and dense skip connections. Semantically high-level features in decoder network fused with low-level semantic features after following deep supervision in skip connection. Deep Residual U-Net (ResUNet) is developed by Zhang et al.^8^ as a combined architecture by utilizing the strengths of U-Net^4^ and deep residual learning^9^. Deep residual learning is proposed to address the problem of degradation in deep convolutional neural networks. Lately, ResUNet++^10^ is developed as a sophisticated version of ResUNet by further utilizing squeeze and excitation blocks^11^, Atrous Spatial Pyramidal Pooling (ASPP)^12^, and attention blocks.

The performance of most of the above architectures are demonstrated by dense predictions in multi-stage Cascaded CNNs. However, this method utilizes excessive and redundant computational cost for feature processing during the segmentation task. Along with the developments to reduce the computational cost in segmentation, researchers have been proposed spatial modules with attention mechanisms to suppress irrelevant features while highlighting the most relevant spatial information for the segmentation task. Attention mechanisms could significantly enhance the extraction of salient features to learn focus target with comparably fewer. Oktay et al.^13^ proposed Attention U-Net architecture based on U-Net and end-to-end-trainable attention module which was proposed for image classification by Jetley et al.^14^. Attention U-Net has implemented attention mechanism in skip connection to extract salient features to fuse with high-level semantic features. It could enhance learning by highlighting important features while suppressing redundant regions for the specific segmentation task. Furthermore, transformer-based attention mechanism^15^ which is popular in Natural Language Processing (NLP) applications, is implemented in ResUNet++ architecture. Squeeze-and-excitation^11^ is developed as a channel attention mechanism using global average pooling to highlight important channels while suppressing channels with minor relevance. This channel attention mechanism is applied in ResUNet++ and other recent developments. Woo et al.^16^ proposed Convolutional Block Attention Module (CBAM) which is a fusion of channel attention mechanism and spatial attention mechanism. CBAM is a lightweight mechanism that is easy to integrate into neural networks and has demonstrated success in recent developments^17–19^. Furthermore, researchers who developed attention mechanisms have emphasized that the accuracy and sensitivity of the prediction greatly improved with the attention mechanisms by utilizing comparatively a smaller number of parameters.

In this paper, we explore the effectiveness of attention mechanisms to improve tumor segmentation performance with less computational cost. Moreover, we investigate the viability of applying these recently developed methods to improve the segmentation of liver tumors with fuzzy boundaries. Inspired by U-Net^4^, deep residual learning^9^, and attention mechanisms^13–16^, we designed a novel deep learning architecture named MANet by following U-Net as the base architecture, which has shown state-of-the-art performance in various biomedical applications. Attention mechanisms are utilized for better channel and spatial information extraction to improve the segmentation performance of the model. The proposed model is trained and evaluated with the publicly available LiTS17 dataset^20^ and 3DIRCADb dataset^21^ under various evaluation metrics. Our experiment results proved that the proposed model is efficient and effective for tumor segmentation compared to baseline architectures of U-Net, Attention U-Net, and U-Net + Resnet18. In summary, the contribution of the Multi Attention Network (MANet) can be shown as follows.

1. We propose a novel MANet architecture of semantic segmentation neural network by utilizing the strengths of residual blocks, channel attention, and spatial attention mechanisms elaborated in CBAM. The attention mechanism is integrated to extract spatial features from the encoder to combine with corresponding high-level semantic features in the decoder, which is proposed in Attention U-Net architecture. MANet has adapted U-Net architecture as a basis for the development.
2. The attention mechanisms implementation in the encoder path, skip connection, and decoder path greatly improved the focus on the region of interest of the target segmentation, achieving the best sensitivity score in all the experiments.

## Related works

Automatic liver tumor segmentation has been a most trending topic in deep learning based medical research field. To improve the quality and accuracy of the diagnosis, deep learning advancements provide significant contribution by delivering supportive and faster opinion for clinical management. The rapid development in machine learning technology in liver tumor diagnosis, has been reached to closer level of radiologists^22, 23^. A computer-aided diagnosis system to detect and grading liver tumors based on multi-phase contrast-enhanced magnetic resonance imaging (CE-MRI) proposed by Alksas et al.^24^. They have explored the effectiveness of imaging markers with machine learning techniques alike support vector machine (SVM), naive bayes classifier (NB), k-nearest neighbors (KNN), and linear discriminant analysis (LDA). Large scale deep learning-based study has conducted with seven types of liver lesions and clinical data^23^. Seven models have experimented for liver lesion categorization task based on enhanced MRI, unenhanced MRI and clinical data. Two models developed based on MRI imaging and clinical data, proved better diagnostic performance compared to experienced radiologists. A deep learning system which is developed for detection of Hepatocellular Carcinoma based on CE-MRI, has indicated similar capability to less experienced radiologist^22^. The architecture is designed based on fine-tuned convolutional neural network (CNN), which is approximately six times faster than human. Multi-phasic MRI based convolutional neural network classifier has developed assess the feasibility of liver lesion classification^25^. Imaging Reporting and Data System (LI-RADS), that radiological experts defined general categorization standards referred to the study. To minimize image interpretation variability, facilitate quality assurance and research development, they suggested to utilize deep learning based automatic categorization standard for systems like LI-RADS.

Apart from the deep learning-based classification, the research field turned to another direction with the development of fully convolutional neural network (FCN)^3^. FCN pixel level classification which could give segmentation output by end-to-end training. U-Net architecture gain popularity in medical research field as the extended development of FCN. There are significant number of extensions based on U-Net architecture^5, 7, 9, 10, 26^, became widely used deep learning architectures in liver and tumor segmentation task. Alirr^27^ proposed a deep learning based automatic liver and tumor segmentation method which is based on U-Net architecture. HU windowing and median filtering are used in preprocessing steps and tensor-based 3D edge enhancing diffusion (EED) filter is used to enhance training data for the training process. Ayalew et al.^28^ explored liver and tumor segmentation method based on U-Net with parameter reduction. Class imbalance method and data refinement techniques are utilized to improve the segmentation performance with less computational cost compared to the original U-Net. U-Net++^7^ is based on nested and dense skip connections and UNet 3+^26^ is the further developed version with deep supervision in each level of the decoder path. Li et al.^29^ upgraded UNet++ architecture by applying a channel attention mechanism to long-hop connections. The implementation of channel attention could reduce the eigenvalue loss. Moreover, multi scale feature extractors with dilated convolution kernels utilized to enhance the feature representation for segmentation task in CE-Net^30^. Lei et al.^31^ presented further development in DefED-Net to enhance feature extraction and representation ability by using deformable convolution to extract variable liver and tumor shape features in different slices, but both networks required more parameters to enhance the performance.

Oktay et al.^13^ presented Attention U-Net which could emerge the popularity in attention mechanisms in liver and tumor segmentation research developments. Attention mechanisms investigate the capability to emphasize important features while suppressing irrelevant features for the segmentation task. Attention UNet++^32^ is utilized the same attention mechanism to improve UNet++ architecture. The developed architecture demonstrated better focus on target regions while suppressing irrelevant areas. Wang et al.^33^ utilized spatial attention gates to emphasize important features for liver segmentation. Residual learning is implemented to improve UNet based attention networks^34–37^. UNet++ network is improved with residual learning and spatial attention to minimize learning errors and improve the semantic gap between features of the encoder and decoder path of the network^34^. Attention mechanisms could significantly minimize the training parameter count of the networks. RA-UNet^38^ presented an attention mechanism that is implemented between the encoder and decoder using the max-pooling operation to highlight important features while reducing the noise. And residual connections are applied to retain original features while emphasizing salient features. Apart from the spatial attention mechanism, channel attention plays an important role in better feature enhancement. Global attention and hybrid attention mechanisms are designed to effectively focus on local and global features of the segmentation^39, 40^. Self-attention based architecture is developed to enhance the feature representation for liver and tumor segmentation^41^. However, the network has not utilized the strengths of the attention mechanisms for the feature recalibration in the encoder path and skip connections. Furthermore, Hu et al.^11^ exploited the inter-channel relationship by the Squeeze-and-excitation (SE) module, which has been developed using global average-pooled features to calculate channel-wise attention. Several deep learning architectures have utilized the SE module to increase its sensitivity to relevant features while suppressing redundant features and achieved success in medical image segmentation^10, 33^. MS-UNet^42^ is designed with the SE module to improve the channel-wise feature recalibration. HFRU-Net^43^ is implemented SE module in skip connection to adaptively recalibrate encoder features to fuse with the deep features. Woo et al.^16^ suggested the improved channel attention using both average-pooling and max-pooling, and proved its effectiveness. Moreover, they proposed Convolutional Block Attention Module (CBAM) which sequentially combined channel attention and spatial attention. Pang et al.^44^ proposed TA-Net utilizing various deep learning techniques like inception blocks, context blocks, and attention blocks to achieve better feature representation to improve performance in medical image segmentation. They have identified channel attention with both average pooling and max pooling outperforms channel attention with only average pooling, where it is used in shallow feature extraction path and deep feature extraction path separately. Zhao et al.^45^ used CBAM by splitting it to channel attention for deep feature extraction at the bottom of the architecture and spatial attention for both encoder and decoder at the top level to emphasize salient features in input-level shallow features, and semantically high-level features in output. Small Attention-UNet (SmaAt-UNet)^46^ is applied CBAM in skip connection and bottleneck of the network. CBAM is utilized to amplify important encoder features to concatenate with deep features in the decoder. The depthwise-separable convolutions have significantly reduced the training parameters of the network. Furthermore, CBAM is utilized to recalibrate channel weights and enhance spatial features at the deeper level of the network in S-Net^47^, which demonstrated significant improvement in liver tumor segmentation.

## Methodology

### MANet architecture

**Figure 1:**
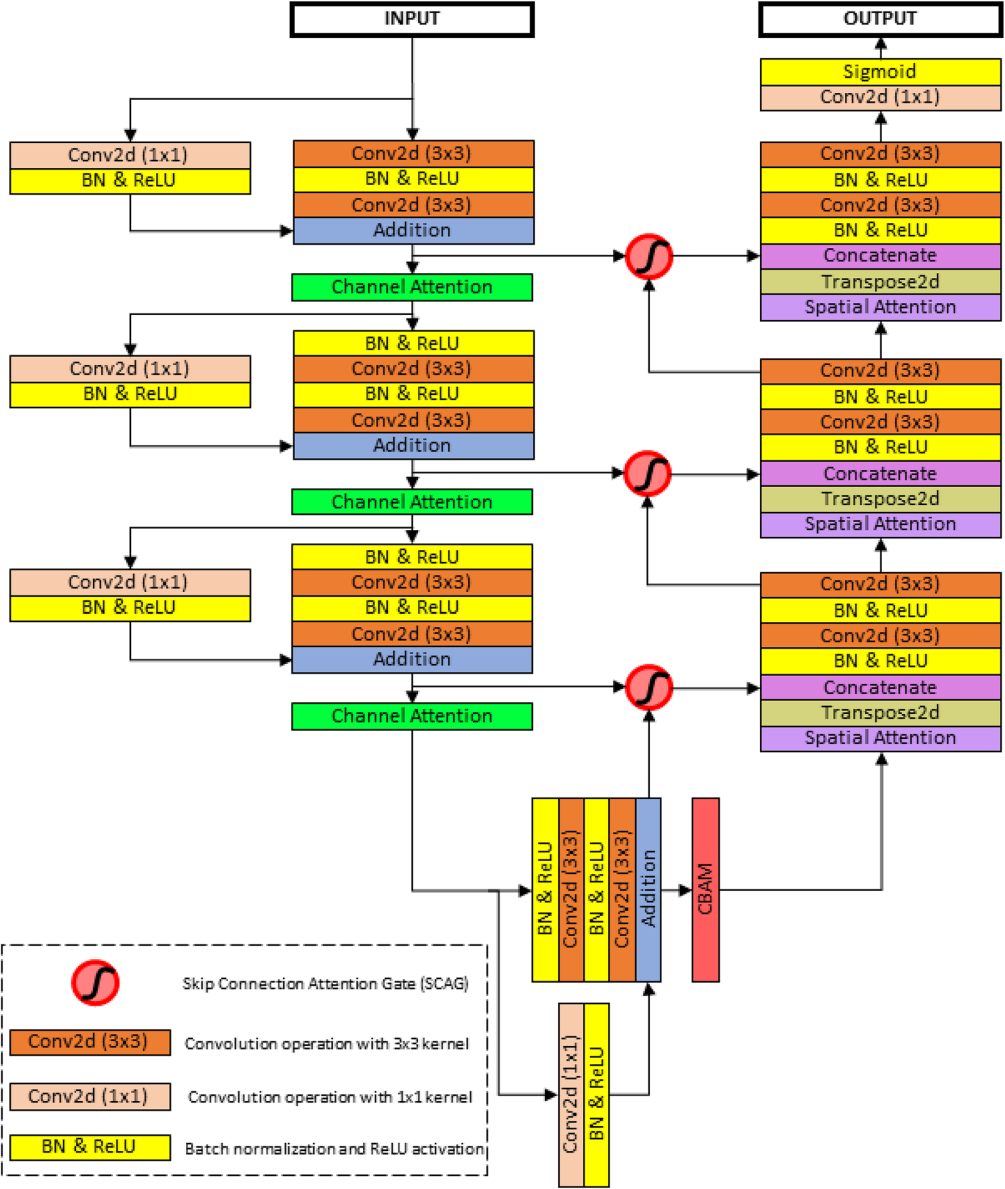
Block diagram of the proposed MANet network architecture.

**Table 1:** The network structure of the proposed MANet architecture..

Block name	Operation	Filter size	Number of filters	Stride	Output size
Input image					512 $\times$ 512 $\times$ 3
Encoder 1	Conv 1	3 $\times$ 3	68	1	512 $\times$ 512 $\times$ 68
	Conv 2	3 $\times$ 3	68	1	512 $\times$ 512 $\times$ 68
Encoder 2	Conv 3	3 $\times$ 3	136	2	256 $\times$ 256 $\times$ 136
	Conv 4	3 $\times$ 3	136	1	256 $\times$ 256 $\times$ 136
Encoder 3	Conv 5	3 $\times$ 3	272	2	128 $\times$ 128 $\times$ 272
	Conv 6	3 $\times$ 3	272	1	128 $\times$ 128 $\times$ 272
Bridge	Conv 7	3 $\times$ 3	544	2	64 $\times$ 64 $\times$ 544
	Conv 8	3 $\times$ 3	544	1	64 $\times$ 64 $\times$ 544
Decoder 1	Conv 9	3 $\times$ 3	136	1	128 $\times$ 128 $\times$ 136
	Conv 10	3 $\times$ 3	136	1	128 $\times$ 128 $\times$ 136
Decoder 2	Conv 11	3 $\times$ 3	68	1	256 $\times$ 256 $\times$ 68
	Conv 12	3 $\times$ 3	68	1	256 $\times$ 256 $\times$ 68
Decoder 3	Conv 13	3 $\times$ 3	68	1	512 $\times$ 512 $\times$ 68
	Conv 14	3 $\times$ 3	34	1	512 $\times$ 512 $\times$ 34
Output	Conv 15	1 $\times$ 1	3	1	512 $\times$ 512 $\times$ 3

We propose the MANet architecture that is evaluated for liver tumor segmentation. The block diagram of the proposed architecture is shown in Fig. 1. The structure of the proposed network is presented in the Table 1. MANet architecture is an improved version of U-Net^4^ by utilizing strengths of deep residual learning^9^ and attention mechanisms^13, 16^. The architecture is designed with an encoder, bridge, and decoder. The encoder propagates information through residual blocks and channel attentions based on the input image. The decoder generates pixel-wise classification to compute semantic segmentation. Feature propagation at the deepest level by residual unit and feature enhancement in the channel and spatial level computed in the bridge.

In general, the deep neural networks suffer from degradation due to error feature learning, we have applied deep residual learning technique to create encoder residual blocks to address the degradation problem in the network. As can be seen in the diagram, the encoder residual block is combined with two convolution layers, batch normalization, and Rectified Linear Unit (ReLU) activation. ReLU activation followed by batch normalization is applied to eliminate the gradient vanishing problem and accelerate the convergence speed of the network. The feature propagation output of each encoder block is directed to the channel attention module to recalibrate channel weights for a better inter-channel relationship to enhance semantic feature extraction. The spatial dimension downsampling operation is performed by applying stride of 2 in the first convolution layer in each residual block. The spatial attention mechanism is applied in skip connection to extract important shallow features with the gate signal, which is extracted by deep features in the lower stage. Lower-stage deep features are up-sampled by transposed convolution and concatenated with corresponding shallow features extracted through the attention mechanism.

The input of the decoder block is passed through spatial attention to emphasize semantic information and retain over decoder path. As in the encoder block, batch normalization and ReLU activation are sequentially applied after each convolution layer in decoder blocks too. Two succussive convolution layers with 3 $\times$ 3 kernels are employed for feature propagation in the decoder block. The output of the decoder path followed through 1 $\times$ 1 convolution and finally applied sigmoid activation to generate the segmentation output.

### U-Net and residual blocks

In semantic segmentation, the fusion of high-resolution low-level features and high-level semantic features is crucial to obtain better segmentation performance^3, 4^. The skip connection in U-Net that is applied in each stage of the network could enhance the segmentation performance, and achieve success in the medical image segmentation field. Utilizing skip connections in each stage of the network facilitates information propagation without degradation, further explained in deep residual learning^9^ which proposed to improve the training errors in deep neural networks. And validated in state-of-the-art approaches^8, 10^. Inspired by residual connections, we designed an encoder with residual blocks which consist of two 3 $\times$ 3 convolution blocks and one residual connection. Due to the memory limitations, a convolution block with a 1 $\times$ 1 kernel is applied to control channels to perform the addition of the input and output of the residual block. Batch normalization and ReLU activation are applied in the residual block including skip connection to alleviate performance degradation, gradient vanishment and accelerate the feature propagation.

### Attention mechanisms

To extract better contextual information, attention mechanisms play a major role in the segmentation task. We proposed a novel MANet inspired by two attention mechanisms proposed in^13, 16^. Attention mechanisms give the capability to enhance feature representations by utilizing a comparatively small number of parameters. Overall, attention mechanisms can split into two categories, Channel attention, and Spatial attention. Channel attention performs global average pooling to calculate the statistical weight of each channel. Spatial attention performs global pooling operations across the channel dimension to extract contextual information. Moreover, channel attention guides the network to focus on “what” salient features to represent while spatial attention explores “where” important features are located in the feature map. The proposed MANet comprises four attention mechanisms named Skip connection attention gate, Channel attention, Spatial attention, and convolutional block attention module (CBAM).

**Figure 2:**
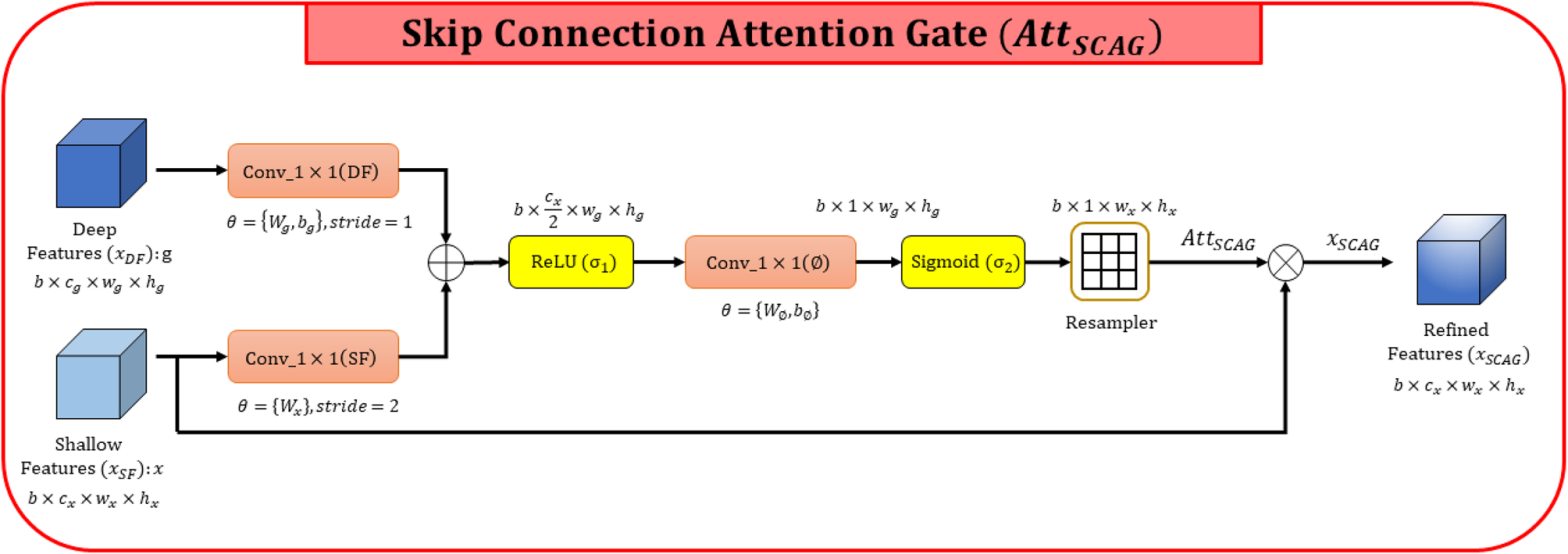
Schematic diagram of Skip Connection Attention Gate (SCAG).

**Skip connection attention gate** is designed to capture important shallow features from the encoder to concatenate with semantically high-level features in the decoder. The attention is computed by aggregating shallow features $x_{SF}$ and deep features $x_{DF}$ in the previous decoder block in the decoder path of the network. The block diagram of the skip connection attention gate is illustrated in Fig. 2, and can be formulated as follows:

1 $$\begin{aligned}&x_{conv\_1 \times 1(SF)} = W_x\cdot x_{SF}, \;\; x_{SF} \in \mathbb {R}^{b\times c_x \times w_x \times h_x}, \;\; x_{conv\_1 \times 1(SF)} \in \mathbb {R}^{b\times \frac{c_x}{2} \times w_g \times h_g}\\&x_{conv\_1 \times 1(DF)} = W_g\cdot x_{DF} + b_g, \;\; x_{DF} \in \mathbb {R}^{b\times c_g \times w_g \times h_g}, \;\; x_{conv\_1 \times 1(DF)} \in \mathbb {R}^{b\times \frac{c_x}{2} \times w_g \times h_g}\\&Att_{SCAG}(x_{SF}, x_{DF};\theta _{SCAG}) = \sigma _2 (W_\phi \cdot \sigma _1 \left( x_{conv\_1 \times 1(SF)} + x_{conv\_1 \times 1(DF)}\right) + b_\phi ) \end{aligned}$$

Where $\sigma _1$ symbolizes the ReLU activation function and $\sigma _2$ denotes the sigmoid activation function to generate the final attention map. The batch size is *b*, *c* is the number of channels and $w\times h$ is the size of the feature maps. The attention mechanism $Att_{SCAG}(x_{SF}, x_{DF};\theta _{SCAG})$ is parameterized by $\theta _{SCAG} = \{W_x,\, W_g,\, b_g,\, W_\phi ,\, b_\phi \}$, weights and bias terms of the convolutions symbolize by *W* and *b* respectively. Input feature maps ($x_{SF}$,  $x_{DF}$) are linearly mapped to half of the shallow feature maps $( \mathbb {R}^{b\times \frac{c_x}{2} \times w_g \times h_g})$ in the dimensional space, formulated in (1). The enhanced feature representation $x_{SCAG}$ is formulated as follows:

2 $$\begin{aligned} x_{SCAG} = x_{SF}\otimes Att_{SCAG}(x_{SF}, x_{DF};\theta _{SCAG}), \;\; x_{SCAG} \in \mathbb {R}^{b\times c_x \times w_x \times h_x} \end{aligned}$$

Where, element-wise multiplication denotes by $\otimes$

**Figure 3:**
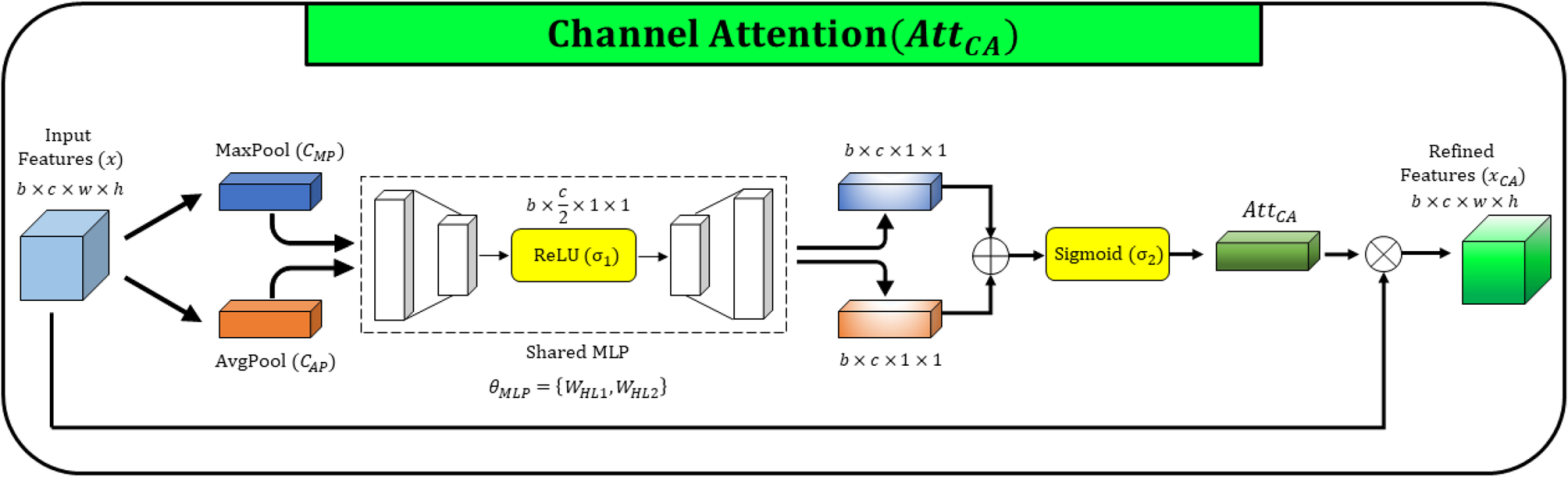
Schematic diagram of Channel Attention (CA).

**Channel attention** captures the inter-channel relationship and recalibrates it to enhance the segmentation performance. The channel attention that comprises both global max pooling and global average pooling demonstrated better performance compared to the “Squeeze and Excitation” channel attention technique which is based on only global average pooling. The fusion of max-pooling and average-pooling features provide a better inter-channel relationship compared only with average-pooling feature extraction to recalibrate the channels^16, 44^. Because of the advantages, we use the channel attention mechanism which is strengthened with both average-pooling and max-pooling operations in the proposed architecture. As illustrated in Fig. 3, input feature maps (*x*) are subjected to global pooling operations to compute the global max pooling feature descriptor $C_{MP}$ and global average pooling feature descriptor $C_{AP}$ $(C_{MP}, C_{AP} \in \mathbb {R}^{b\times c \times 1 \times 1})$.

3 $$\begin{aligned}&C_{MP} = MaxPool(x)\\&C_{AP} = AvgPool(x) \end{aligned}$$

The output of the global polling operations is directed to capture channel-wise correlation by shared multi-layer perceptron (MLP). *MLP* is designed with two hidden layers and ReLU, formulated in (5). To reduce the parameter count, the output size of the first hidden layer is set to half of the input channels, i.e., $\mathbb {R}^{b\times \frac{c}{2} \times 1 \times 1}$. Next, sigmoid activation is applied to the summation of feature descriptors computed from *MLP*.

4 $$\begin{aligned} Att_{CA}(x; \theta _{CA}) = \sigma _2(MLP(C_{MP})+ MLP(C_{AP})) \end{aligned}$$

Where, *MLP* is formulated as follows,

5 $$\begin{aligned} MLP(x; \theta _{MLP}) = W_{HL2} \cdot \sigma _1 (W_{HL1} \cdot x) \end{aligned}$$

The channel attention mechanism is formulated in (4), where $\sigma _1$ and $\sigma _2$ denote ReLU and sigmoid activation functions respectively. The parameters of the channel attention refer to $\theta _{CA} = \theta _{MLP} = \{W_{HL1}, W_{HL2}\}$, which are utilized in two hidden layers in *MLP*. After the channel attention computation, calibrated feature representation $x_{CA}$ is calculated by the element-wise multiplication as follows.

6 $$\begin{aligned} x_{CA} = x \otimes Att_{CA}(x; \theta _{CA}), \;\; x_{CA} \in \mathbb {R}^{b\times c \times w \times h} \end{aligned}$$

**Figure 4:**
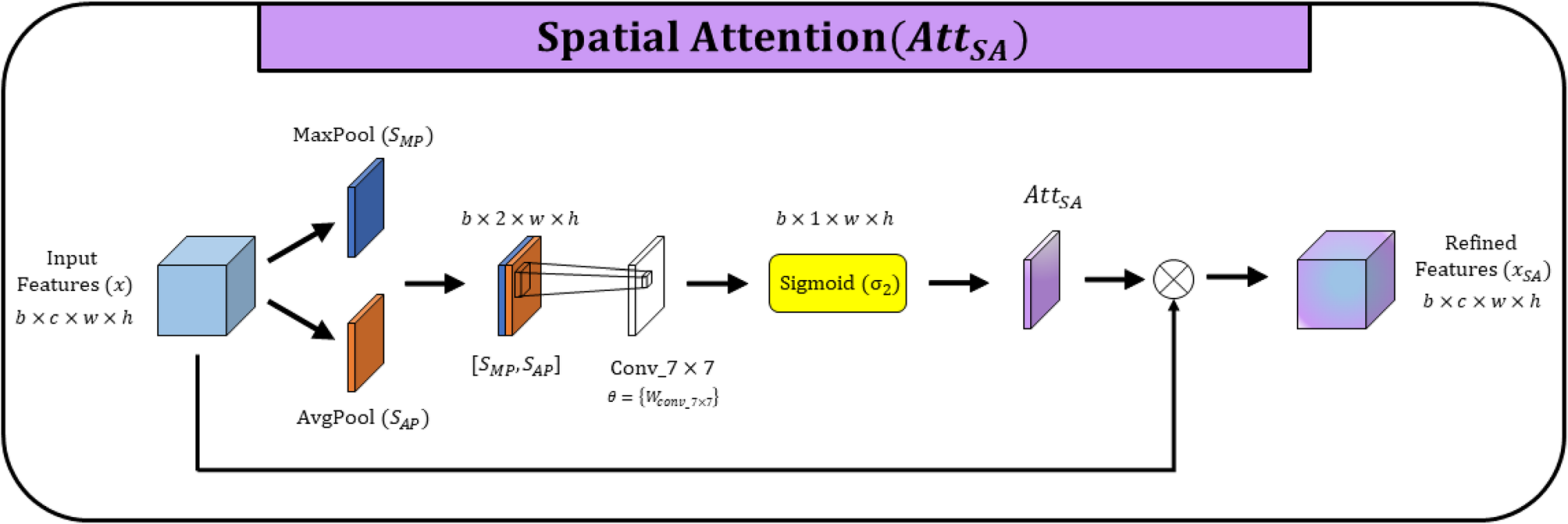
Schematic diagram of spatial attention (SA).

**Spatial attention** is design to capture important spatial features to enhance segmentation performance. Spatial attention mechanism is applied to decoder block to leverage important tumor features while suppression non-tumor features in decoder path. As shown in Fig. 4, global max pooling an average pooling are performed along with the channel axis for the input features (*x*) to calculate spatial feature descriptors $S_{MP}, S_{AP} \in \mathbb {R}^{b\times 1 \times w \times h}$ respectively. After that, both spatial feature mas are concatenated. That feature maps with two channels represent the contextual tumor feature aggregation across the spatial locations. Convolution operation with $7\times 7$ kernel is perform to further extract important contextual information which are highly relevant to segment liver tumors. The sigmoid activation function $(\sigma _2)$ is applied to the spatial attention map generated by the 7 $\times$ 7 convolution layer. Spatial attention mechanism is formulated as follows.

7 $$\begin{aligned} Att_{SA}(x; \theta _{SA}) = \sigma _2(W_{conv\_7 \times 7} \cdot ([S_{MP}, S_{AP}])) \end{aligned}$$

Where concatenation of global max pooling and average pooling denotes by $([S_{MP},S_{AP}])$ and $\theta _{SA}=\{W_{conv\_7 \times 7}\}$ refers to the parameters of the convolution operation in the spatial attention mechanism. Spatial attention map generated by $Att_{SP} (x; \theta _{SP})$, element-wise multiplied with input feature maps to recalibrate features corresponding to the attention feature map. The enhanced feature representation $x_{SA}$ formulated as shown below.

8 $$\begin{aligned} x_{SA} = x \otimes Att_{SA}(x; \theta _{SA}), \;\; x_{SA} \in \mathbb {R}^{b\times c \times w \times h} \end{aligned}$$

**Figure 5:**
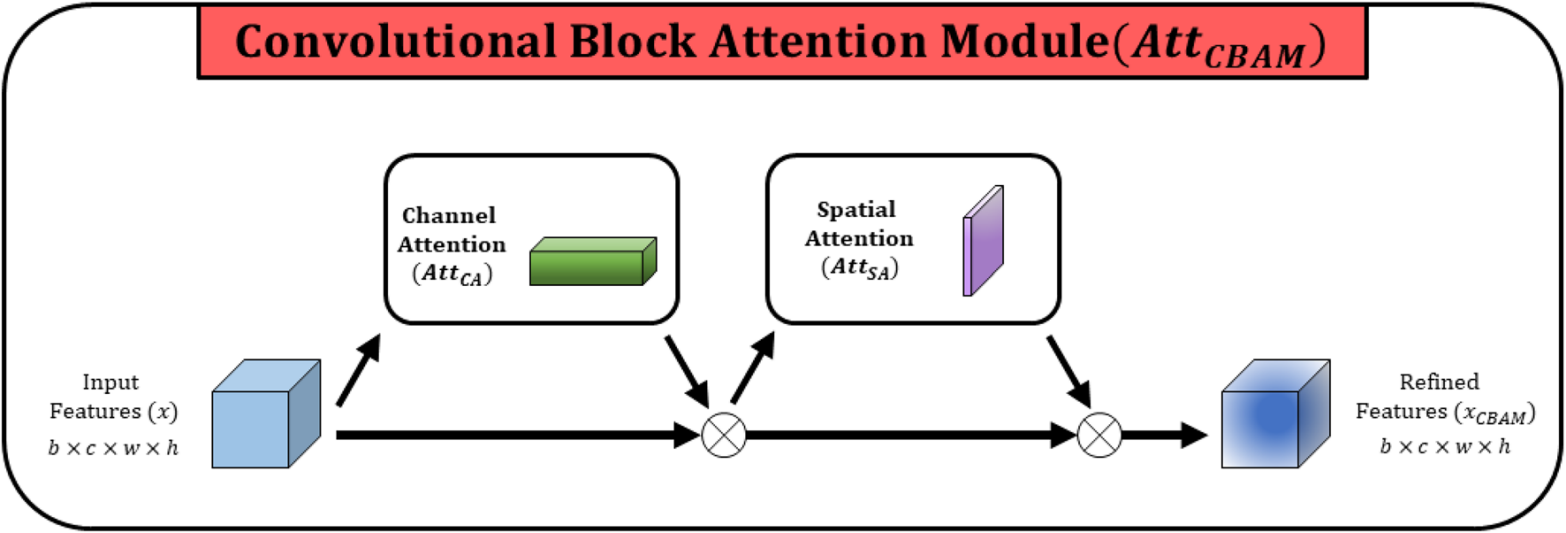
Schematic diagram of Convolutional Block Attention Module (CBAM).

**Convolutional block attention module (CBAM)** is designed by sequentially applying 1D channel attention and 2D spatial attention in the convolution neural network as illustrated in Fig. 5. In the proposed network, CBAM is applied in the bridge which is connecting encoder path and the decoder path of the network. At the deepest level of the network, CBAM is employed to extract important features in channel dimension and capture spatial feature representations in spatial dimension sequentially to enhance segmentation performance. The calibrated feature maps $x_{CBAM}$ from the convolutional block attention mechanism is formulated in (9), which is based on channel attention and spatial attention formulated in (4) and (7) respectively.

9 $$\begin{aligned}&x_{CBAM} = x \otimes Att_{CA}(x; \theta _{CA}) \otimes Att_{SA}(x \otimes Att_{CA}(x; \theta _{CA}); \theta _{SA})\\&x_{CBAM} \in \mathbb {R}^{b\times c \times w \times h} \end{aligned}$$

## Experimental setup

### Dataset and preprocessing

The proposed method is evaluated on the public dataset of MICCAI 2017 Liver Tumor Segmentation (LiTS17) challenge^20^ and 3DIRCADb dataset^21^. The LiTS dataset consist of 131 CT scans for training and 70 CT scans for testing, which have been collected from several clinical sites from different regions of the world. The dataset contains diverse types of liver tumor diseases and acquired with different CT scanners. LiTS dataset has been provided segmentation masks for liver and tumors only for the training set which consists 131 CT scans. Segmentation masks annotated by trained radiologists at each clinical sites and further verified by three experienced radiologists. Moreover, CT scan volume contains slices in range from 42 to 1026, where the image size is $512 \times 512$. The 3DIRCADb dataset contains 20 CT volumes with liver tumors in 15 CT volumes. 20 CT volumes of the 3DIRCADb dataset are included in the LiTS dataset (from volume 28 to volume 47)^39^. The number of tumors in the scan varies between 0 and 75, size of the tumors varies between $38 \; {\text {mm}}^{3}$ and $349 \; {\text {cm}}^{3}$.

The CT scan slices represent different organs and regions by a wide range of intensity values which varies in between -1000 and 3000. To enhance the liver area from the abdominal scan, image intensities of all the scans are truncated to the range of [−150, 250] Hounsfield Unit (HU) and followed histogram equalization and normalization before feeding to the training process. To evaluate the performance of the proposed liver tumor segmentation method, CT slices with tumor annotations are selected for the experiments. One scan is excluded from the experimental data set due to abnormality, a total of 130 CT scans (7050 slices) have been considered for the experiments. We have conducted experiments based on slices and volumes to evaluate the models. All the data randomly split into 4:1 ratio for the training set and test set. In the slice-based experiment, all the slices randomly split into 5640 slices for the training set and 1410 slices for test set. And all the scans randomly split into 104 volumes (5408 slices) for the training set and 26 volumes (1642 slices) for the test set to conduct the volume-based experiment. The training set and test set for the volume-based experiment include 8 and 7 volumes, respectively, of the 3DIRCADb dataset. We have not split the dataset into a validation set due to the limited amount of biomedical data.

To minimize the risk of overfitting, we have used real-time data augmentation with Albumentations^48^, which randomly transform the batch of the data without increasing number of slices. The random transformations provide no duplication data among training process in different epochs. Vertical flip, shift, scale, rotate operations are applied during the random transformations.

### Implementation details

**Figure 6:**
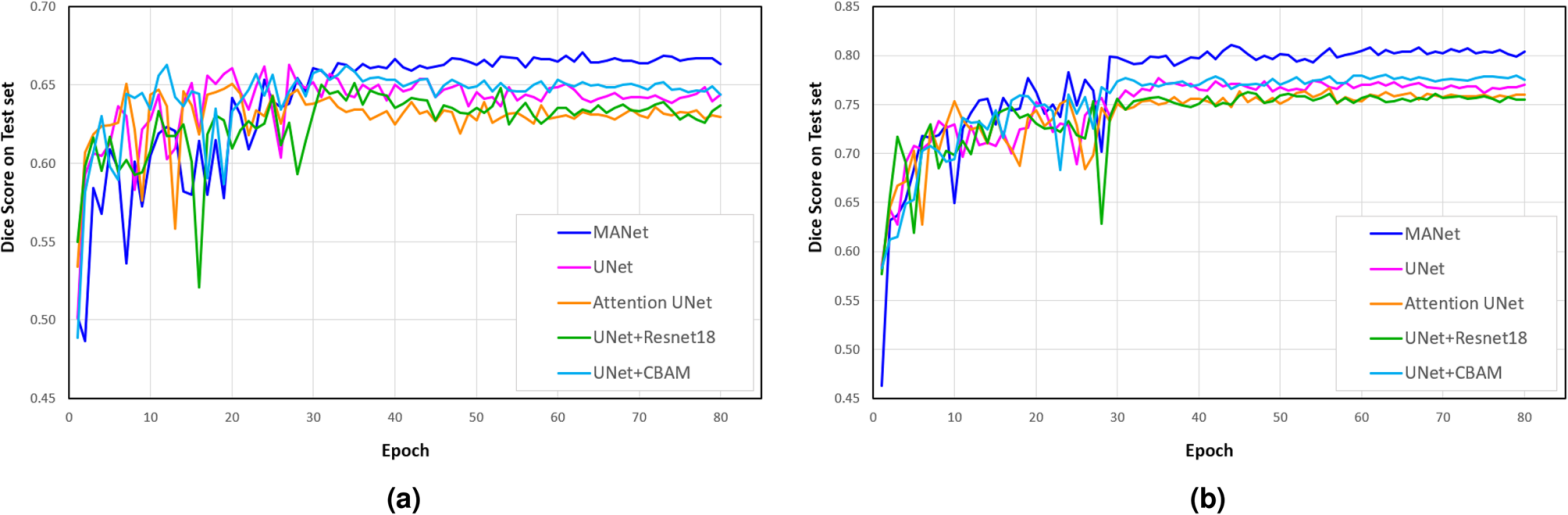
The baseline models and proposed model evaluation of Dice score during the 80 epochs of training on test set. (**a**) Volume-based segmentation performance. (**b**) Slice-based segmentation performance.

We run all the experiments on a workstation with Windows 11 operating system, RTX2070 GPU with 8 GB memory, 32 GB of RAM, Intel(R) Core (TM) i7-9750H CPU @ 2.60GHz 2.59 GHz (6 cores), and PyTorch 1.9 deep learning framework for implementation. In the training phase, the initial value of the learning rate is set to 0.0001 and is attenuated by 0.1 in every 30 epochs. All the experiment networks are trained for 80 epochs to ensure the model convergence and best performance for the test set (Fig. 6). The model weights that resulting the highest dice score on the test set during the training process are selected to conduct the model evaluations. Adam optimizer is used to optimize the objective function. The batch size is empirically set to 4 by considering the memory capacity of the GPU. Moreover, we employ the Dice loss function which is a famous loss function in medical image segmentation to optimize the training process of the proposed MANet.

### Evaluation metrics

To effectively evaluate the tumor segmentation performance of the experimental models, the seven most popular evaluation metrics are calculated. In general, selected evaluation metrics can be categorized into two sections: overlap-based methods, and boundary-distance-based methods. Dice score (DICE also known as F1 score) is one of the most frequently used evaluation metrics, Jaccard index is known as intersection over union (IoU), volume overlap error (VOE) is the corresponding error metric for the Jaccard index (1 - Jaccard index), accuracy, sensitivity (recall), specificity are denoted as overlap-based methods. The average symmetric surface distance (ASSD) is calculated average distance from points in the predicted binary mask and the ground truth binary mask, is denoted as a boundary-distance-based method^49^. The evaluation metrics are formulated as shown below:

$$\begin{aligned}&DICE = \frac{2 |A \cap B|}{|A| + |B|} = \frac{2TP}{2TP + FP + FN} \\&Jaccard \; index = IoU = \frac{|A \cap B|}{|A \cup B|} = \frac{TP}{TP + FP + FN} \\&VOE = 1 - \frac{|A \cap B|}{|A \cup B|} = 1 - Jaccard \; index \\&Accuracy = \frac{TP+TN}{TP+TN+FP+FN} \\&Sensitivity \; (Recall) = \frac{TP}{TP+FN} \\&Specificity = \frac{TN}{TN+FP} \\&ASSD = \frac{\sum _{x \in \partial B} d(x,\partial A) + \sum _{y \in \partial A} d(y,\partial B)}{|\partial B|+|\partial A|} \end{aligned}$$

Where the predicted binary mask and ground truth binary mask are denoted by A and B respectively. TP, TN, FP, and FN represent the pixel count of true positives, true negatives, false positives, and false negatives respectively. The quantitative performance of the baseline models and the proposed model is shown in Table 2 and Table 3. The performance comparison with state-of-the-art methods is shown in Table 4.

## Results and discussion

This section provides the quantitative and qualitative analysis by comparing the proposed model and the baseline methods. Further evaluation is conducted with state-of-the-art methods to prove the effectiveness and robustness of the proposed network. The ablation study is conducted to evaluate the effectiveness of the architectural design of the proposed network. Moreover, we further conducted a detailed comparison of the number of parameters and the computational cost among all the compared models and the future direction of the research. The proposed architecture can be considered as 7 blocks architecture that is strengthened with attention mechanisms and residual blocks. The depth of the architecture is limited to four to minimize the parameter count and the complexity of the model. To make a fair comparison, we have utilized baseline architectures of Attention UNet, UNet+Resnet18, and UNet+CBAM, which are having the same depth as same as the proposed architecture. And the traditional UNet architecture depth is 5, which is the basis for the proposed architecture and other baseline architectures in the experiments. We have referred to original papers and the codes of the baseline architectures to conduct the experiments. Furthermore, UNet+Resnet18 is UNet architecture with Resnet18 backbone, since the encoder of the proposed architecture is designed with residual blocks. UNet+CBAM is UNet architecture integrated with CBAM where the stages of proposed architecture are designed with CBAM and the submodules (channel attention: $Att_{CA}$ and spatial attention: $Att_{SA}$) of it.

We have evaluated the effectiveness and robustness of the proposed network with other state-of-the-art methods. All the comparison networks are based on UNet architecture. UNet 3+^26^ is the latest development based on UNet architecture in the comparison models. Other comparison networks are developed utilizing the strengths of attention mechanisms and multi-level feature extractors. ResUNet++^10^, SmaAt-UNet^46^, and TA-Net^44^ are the other comparison architectures used to compare the performance of the proposed network.

### Quantitative analysis of segmentation performance

**Table 2:** The quantitative performance comparison on five methods based on UNet for volume-based segmentation and slice-based segmentation experiments (mean ± standard deviation) on the LiTS dataset.

Task	Method	Dice score	ASSD	Jaccard index (IoU)	VOE	Accuracy	Sensitivity (Recall)	Specificity
Volume-based Segmentation	Net	0.6612 ± 0.277	1.0843 ± 1.425	0.5469 ± 0.266	0.4530 ± 0.266	**0.9950** ± **0.004**	0.6394 ± 0.285	0.9987 ± 0.002
	Attention UNet	0.6505 ± 0.278	1.2551 ± 1.338	0.5356 ± 0.263	0.4643 ± 0.263	0.9945 ± 0.006	0.6250 ± 0.292	0.9984 ± 0.002
	UNet + Resnet18	0.6560 ± 0.281	**0.9321** ±** 0.960**	0.5433 ± 0.268	0.4566 ± 0.268	**0.9950** ± 0.005	0.6108 ± 0.294	**0.9991** ±** 0.001**
	UNet + CBAM	0.6635 ± 0.271	1.2795 ± 1.638	0.5487 ± 0.261	0.4512 ± 0.261	0.9946 ± 0.005	0.6678 ± 0.283	0.9981 ± 0.002
	MANet (Proposed model)	**0.6735** ±** 0.267**	1.2049 ± 1.356	**0.5590 **± **0.258**	**0.4409 **± **0.258**	**0.9950** ±** 0.004**	**0.7426 **± **0.283**	0.9978 ± 0.002
Slice-based Segmentation	UNet	0.7790 ± 0.208	0.9009 ± 1.020	0.6744 ± 0.217	0.3255 ± 0.217	0.9940 ± 0.006	0.7476 ± 0.237	0.9982 ± **0.001**
	Attention UNet	0.7676 ± 0.195	0.9188 ± 0.783	0.6550 ± 0.208	0.3449 ± 0.208	0.9935 ± 0.006	0.7423 ± 0.231	0.9978 ± 0.002
	UNet + Resnet18	0.7686 ± 0.211	1.0037 ± 1.429	0.6619 ± 0.223	0.3380 ± 0.223	0.9934 ± 0.007	0.7342 ± 0.245	**0.9984** ±** 0.001**
	UNet + CBAM	0.7784 ± 0.202	0.8241 ± 0.810	0.6720 ± 0.214	0.3279 ± 0.214	0.9941 ± 0.005	0.7439 ± 0.234	0.9982 ± 0.002
	MANet (Proposed model)	**0.8145 **±** 0.150**	**0.7084** ±** 0.701**	**0.7084** ± **0.171**	**0.2915** ± **0.171**	**0.9947** ± **0.004**	**0.8723 **±** 0.173**	0.9970 ± 0.002

The best values are in bold.

**Table 3:** The quantitative performance comparison on five methods based on UNet for volume-based segmentation experiment (mean ± standard deviation) on the 3DIRCADb dataset.

Methods	Dice score	ASSD	Jaccard index (IoU)	VOE	Accuracy	Sensitivity (Recall)	Specificity
UNet	0.5767 ± 0.282	1.2578 ± 1.199	0.4534 ± 0.246	0.5466 ± 0.246	0.9942 ± 0.006	0.4813 ± 0.253	0.9996 ± 0.001
Attention UNet	0.5863 ± 0.281	1.4189 ± 1.288	0.4629 ± 0.245	0.5371 ± 0.245	0.9943 ± 0.006	0.4954 ± 0.259	0.9995 ± 0.001
UNet + Resnet18	0.5941 ± 0.270	**1.2051** ± ** 1.038**	0.4681 ± 0.241	0.5319 ± 0.241	0.9944 ± 0.006	0.4956 ± 0.256	**0.9997** ± **0.001**
UNet + CBAM	0.5763 ± 0.278	1.5157 ± 1.458	0.4521 ± 0.246	0.5479 ± 0.246	0.9941 ± 0.006	0.4909 ± 0.257	0.9995 ± 0.001
MANet (Proposed model)	**0.6400** ±** 0.279**	1.3492 ± 1.362	**0.5227** ± **0.258**	**0.4773 ** ± **0.258**	**0.9947** ± **0.006**	**0.6240** ± **0.298**	0.9990 ± 0.002

The best values are in bold.

We have evaluated and compared the segmentation performance of the proposed MANet under various evaluation metrics which are commonly used in liver tumor segmentation quantitative analysis. The model performances in terms of dice score on the test set during the training phase are plotted as shown in Fig. 6. The evaluation results demonstrate that our proposed MANet is superior in both volume-based segmentation and slice-based segmentation experiments compared to baseline models under most of the evaluation metrics as shown in Table 2. In particularly, the proposed MANet shows improvement in average dice score by more than 3% in slice-based segmentation and 1% in volume-based segmentation while demonstrating almost the same performance gap in the Jaccard index. UNet+CBAM performed almost closer to the proposed model in volume-based segmentation (i.e., lower than MANet by 1% in dice score and Jaccard index) but the proposed MANet could uplift the performance in slice-based segmentation in terms of dice score and Jaccard index around 3% as a percentage. Moreover, MANet achieved minimum volume overlap error (VOE) in both experiments by reaching the highest overlapping rate, which also can be considered as the error metric of the Jaccard index. In terms of ASSD, the proposed model was better compared to baseline models and further validated the highest overlapping rate and its superiority in liver tumor segmentation. Attention UNet has not demonstrated a significant performance boost compared to the based model of UNet. Nevertheless, it could maintain almost the same segmentation performance as UNet+Resunet18 with comparatively less parameter overhead. The proposed model could not attain the best performance in specificity which can be explained by two perspectives. We noticed that some ground truth mask regions were smaller than actual tumor regions and the proposed model could segment and recognize tumor boundaries more accurately, according to the verification of an experienced radiologist in our research team. In terms of evaluation metrics, those particular cases are regarded as false positives (over-segmentation) that lead to diminished specificity. Other than that, over-segmentation can be occurred due to limited parameters in the model (almost the half of parameters compared to the base model UNet). It has been proven by UNet+Resunet18 achieving the best specificity in both slice-based and volume-based segmentation experiments containing the highest count of parameters among all the experimental models. It is worth highlighting that the proposed model has outperformed all the baseline models in terms of sensitivity with a significant performance margin (improved by around 8% in volume-based segmentation and 13% in slice-based segmentation) in both experiments. In particular, comparing the performance of the proposed model and UNet+CBAM, it can be deduced that exploiting channel attention in encoder blocks and spatial attention in decoder blocks is more effective than utilizing CBAM in the encoder and decoder blocks. Moreover, the proposed model demonstrated a significant performance boost compared to Attention UNet by applying attention mechanisms to extract features in all the stages of the network (i.e., encoder, decoder, skip connection) while Attention UNet extracts features in only skip connection by attention gates.

The proposed MANet architecture is further evaluated with the 3DIRCADb dataset which can prove the generalization of the network (Table 3). The proposed network has achieved almost 5% of a performance boost in terms of dice score. It can be demonstrated by achieving minimum volume overlap error (VOE) by reaching the highest overlapping rate. It is worth noting that the sensitivity of the network has maintained a significant gap (around 13%) even in the 3DIRCADb dataset. The volume-based experiment results with both LiTS and 3DIRCADb datasets demonstrated a clear correlation in all the evaluation metrics. However, the proposed network could demonstrate a significant performance boost compared to the comparison networks in terms of dice score and sensitivity in the 3DIRCADb dataset. The proposed network has demonstrated superior performance in both LiTS and 3DIRCADb datasets that can prove the better generalizability of the network.

**Table 4:** The quantitative performance comparison on other state-of-the-art methods for slice-based segmentation experiment (mean ± standard deviation) on the LiTS dataset.

Methods	Dice score	ASSD	Jaccard index (IoU)	VOE	Accuracy	Sensitivity (Recall)	Specificity	Total training parameters (M)
UNet 3+^26^	0.5036 ± 0.341	1.3994 ± 1.857	0.4054 ± 0.306	0.5946 ± 0.306	0.9893 ± 0.010	0.4696 ± 0.364	0.9977 ± 0.005	26.98
ResUNet++^10^	0.8101 ± 0.175	1.0323 ± 0.950	0.6727 ± 0.191	0.3273 ± 0.191	0.9937 ± 0.006	0.8330 ± 0.205	0.9968 ± 0.003	4.06
SmaAt-UNet^46^	0.7880 ± 0.185	0.8300 ± 0.955	0.6802 ± 0.202	0.3198 ± 0.202	0.9938 ± 0.007	0.7433 ± 0.218	0.9986 ± 0.002	4.03
TA-Net^44^	0.7904 ± 0.172	0.9331 ± 0.974	0.6799 ± 0.190	0.3202 ± 0.190	0.9937 ± 0.007	0.7751 ± 0.209	0.9979 ± 0.003	29.57
MANet (Proposed model)	**0.8145** ± **0.150**	**0.7084** ±** 0.701**	**0.7084** ± **0.171**	**0.2915** ± **0.171**	**0.9947** ± **0.004**	**0.8723** ± **0.173**	**0.9970** ± **0.002**	**7.83**

The proposed model results are in bold.

In order to evaluate the robustness and effectiveness of the proposed network, the slice-based segmentation experiment is conducted to compare it with other state-of-the-art methods (Table 4). The latest UNet based development of UNet 3+, ResUNet++, SmaAt-UNet, and TA-Net are used to compare the performance with the proposed MANet architecture. The experimental result of the state-of-the-art methods comparison is shown in Table 4. We can see the proposed MANet architecture demonstrated superior performance in most of the evaluation metrics among other state-of-the-art methods. It is worth mentioning that the architectures with attention mechanisms demonstrated comparatively better performance in the experiment. ResUNet++ has reached closer to the proposed MANet architecture in dice score, however, it has not proven significant performance similarity in sensitivity. Even though ResUNet++ and SmaAt-UNet could achieve better dice scores with comparatively lower parameter overhead, the proposed MANet architecture has proven its superiority with the highest overlapping rate that can be indicated by VOE, ASSD, and Jaccard index.

**Figure 7:**
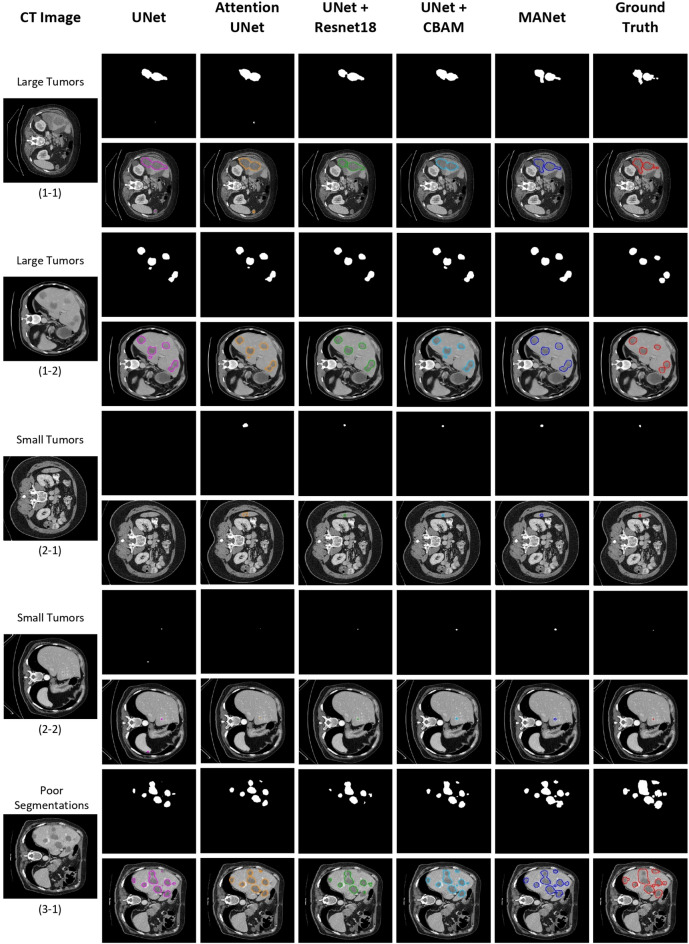
Qualitative analysis of sample segmentation generated by comparison models from the slice-based segmentation experiment. The contour image of the segmentation is illustrated right below the binary segmentation mask. From left to right: the original CT image, results obtained by UNet (pink), Attention UNet (orange), UNet+Resnet18 (green), UNet+CBAM (cyan), MANet (blue), and the corresponding ground truth mask (red). Here, we have illustrated five different samples under three perspectives, which are large tumors, small tumors, and poor segmentation respectively.

**Figure 8:**
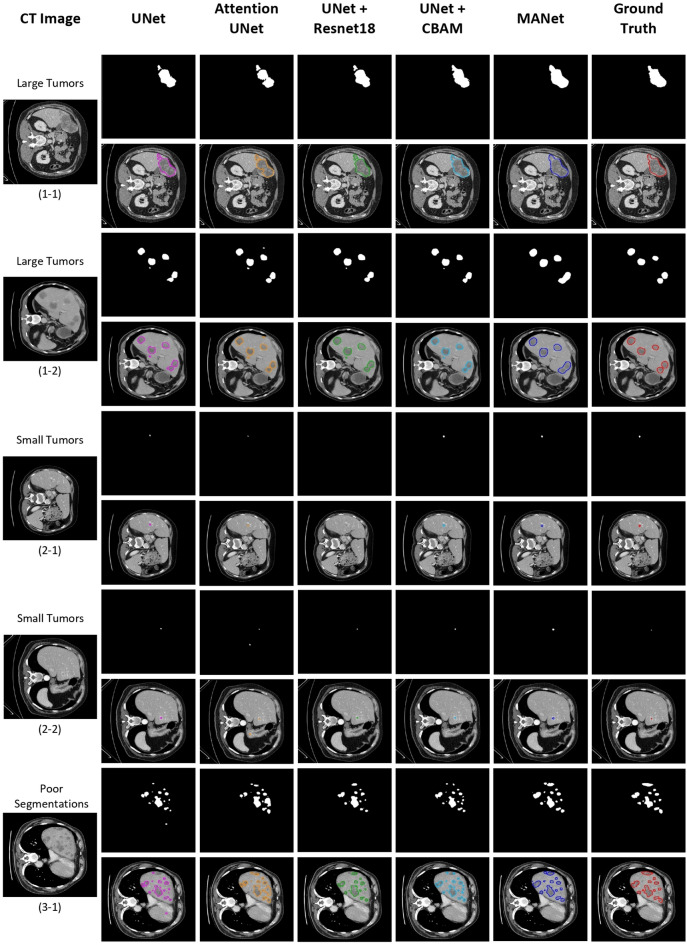
Qualitative analysis of sample segmentation generated by comparison models from the volume-based segmentation experiment. The contour image of the segmentation is illustrated right below the binary segmentation mask. From left to right: the original CT image, results obtained by UNet (pink), Attention UNet (orange), UNet+Resnet18 (green), UNet+CBAM (cyan), MANet (blue), and the corresponding ground truth mask (red). Here, we have illustrated five different samples under three perspectives, which are large tumors, small tumors, and poor segmentation respectively.

**Figure 9:**
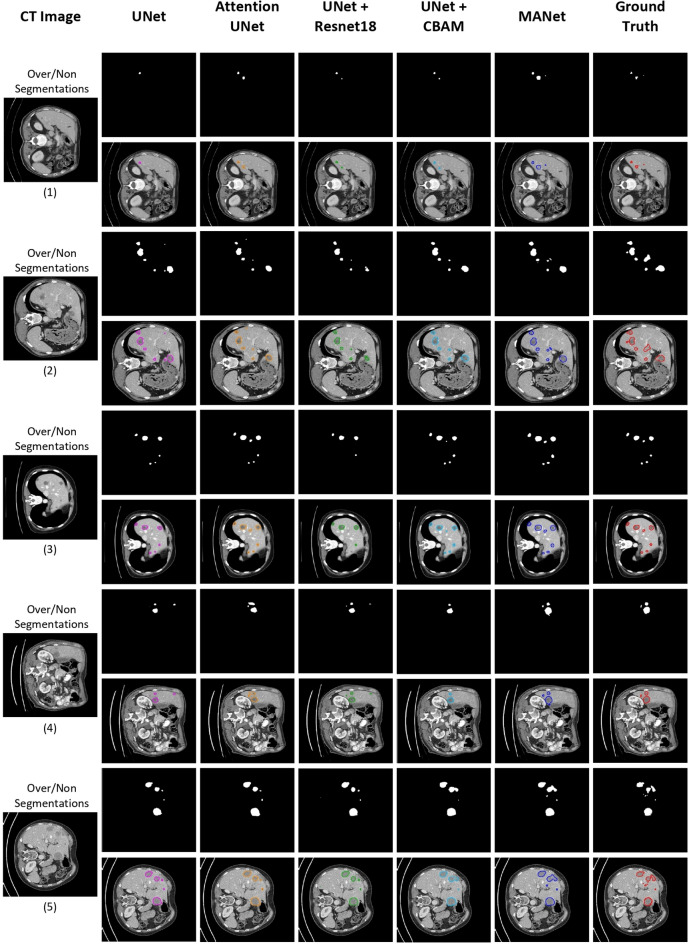
Qualitative analysis of over/non-segmentations in multiple tumor cases generated by comparison models from the slice-based segmentation experiment. The contour image of the segmentation is illustrated right below the binary segmentation mask. From left to right: the original CT image, results obtained by UNet (pink), Attention UNet (orange), UNet+Resnet18 (green), UNet+CBAM (cyan), MANet (blue), and the corresponding ground truth mask (red). Here, we have illustrated five different samples with variable sizes of tumors in multiple tumor cases.

**Figure 10:**
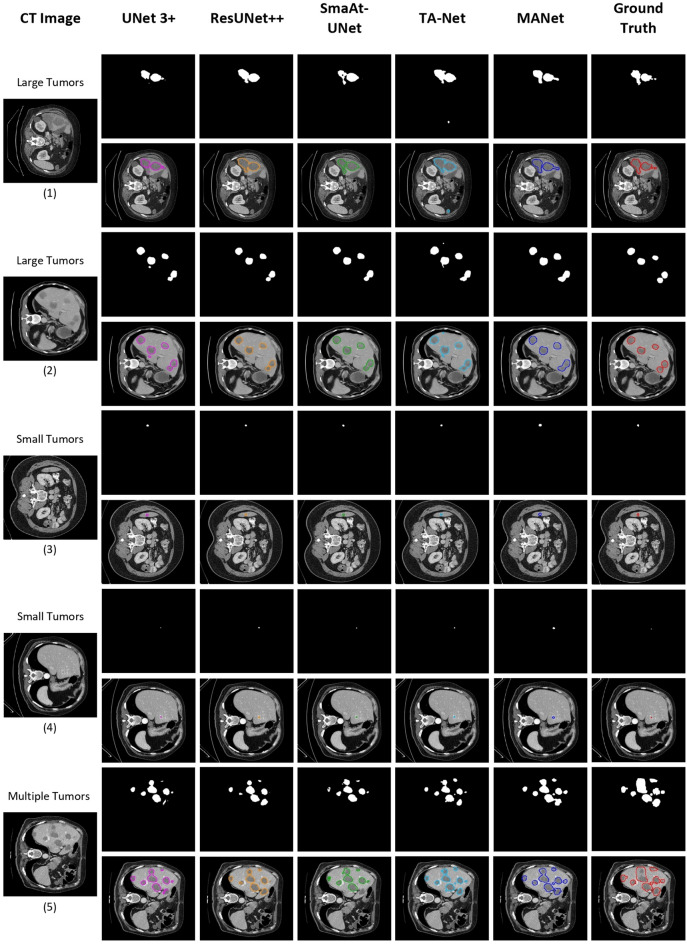
Qualitative analysis of sample segmentation generated by state-of-the-art models from the slice-based segmentation experiment. The contour image of the segmentation is illustrated right below the binary segmentation mask. From left to right: the original CT image, results obtained by UNet 3+^26^ (pink), ResUNet++^10^ (orange), SmaAt-UNet^46^ (green), TA-Net^44^ (cyan), MANet (blue), and the corresponding ground truth mask (red). Here, we have illustrated five different samples under three perspectives, which are large tumors, small tumors, and multiple tumors respectively.

### Qualitative analysis of segmentation mask

The qualitative analysis is also important to evaluate the proposed model performance and feasibility for the tumor segmentation task. We have conducted qualitative analysis by categorizing segmentations into four sections: large tumors, small tumors, poor segmentations, and over/non-segmentations. The volume-based segmentation samples are illustrated in Fig. 8 and slice-based segmentation samples are illustrated in Figs. 7 and 9. The proposed network is further compared with the state-of-the-art models by slice-based segmentation, shown in Fig. 10.

All the models could segment the large tumors with acceptable accuracy. However, most of the baseline models give partial tumor segmentation in the first large tumor sample in both slice-based segmentation and volume-based segmentation, while the proposed model accurately predicts tumor region (see Figs. 7(1-1) and 8(1-1)). The second large tumor case in Figs. 7(1-2) and 8(1-2), is segmented by combining two large tumors into one segmentation blob which recognizes as tumor segmentation with false positives. That combination may be occurred due to the fuzzy boundary of the tumor and roughly similar prediction appeared in baseline models segmentation mask for that particular two large tumors. However, some over-segmentation blobs appeared in baseline models except UNet+Resunet18 in slice-based segmentation but that slight over-segmentation commonly appeared in volume-based segmentation in all baseline methods (see Figs. 7(1-2) and 8(1-2)). Yet, the proposed MANet could maintain similar predictions in both slice-based and volume-based experiments for the same sample. In general, small tumor segmentation is highly challenging not only for automated systems but also experienced radiologists. However, small tumor recognition is crucial to detect the disease in the earlier stage. Some of the baseline models could not give precise and stable segmentation for small tumors while the proposed MANet outperform all the baseline models with stable segmentation (see Figs. 7(2-1, 2-2) and 8(2-1, 2-2)). We should note that two samples were selected under the large tumor and small tumor categories, which were included in the test set in both slice-based and volume-based experiments (i.e., Figs. 7(1-2, 2-2) and 8(1-2, 2-2)), have proven the robustness of the proposed model segmentation in both experiments. Moreover, UNet+CBAM which has exploited attention in each stage of the network as same as proposed MANet, demonstrated almost similar segmentation performance in most of the cases in Figs. 7 and 8. The poor segmentation prediction has illustrated the failure to imitate ground truth and miss segmentations (see Figs. 7(3-1) and 8(3-1)). In that case, the proposed MANet could segment all the tumor regions with comparatively less edge precision while baseline models fail to capture all the tumor regions in the prediction.

Recognizing all the tumors in the CT image is an important aspect of accurate clinical management. To evaluate the proposed model capability, visualized the segmentation performance in multiple tumor cases as shown in Fig. 9. In this illustration we could observe that all the models could segment large tumors with acceptable accuracy and edge precision. However, most of the baseline models unable to segment small tumors in the sample CT images. In particular, UNet and UNet+Resnet18 mostly show partial or missing segmentations for small tumors. The baseline models with attention mechanisms (i.e., Attention UNet and UNet+CBAM) could capture all the tumors in some cases with or without good edge precision which is almost similar to the segmentation of the proposed model (see Fig. 9(2, 3)). Moreover, we noticed that the segmentation of the proposed model in Fig. 9(2) is comparatively poor among all five samples, yet the proposed model segmentation is better compared to the baseline methods. In short, we conclude that the attention mechanism implementation in the proposed MANet architecture is more effective than baseline methods in order to deliver accurate and stable liver tumor segmentation.

In order to make a fair comparison with the state-of-the-art methods, we have visualized the same samples that were visualized in slice-based segmentation in Fig. 7. The segmentation performance of the state-of-the-art models is illustrated in Fig. 10. All the state-of-the-art models demonstrated almost similar performance in small tumor segmentation. However, TA-Net and UNet 3+ models performed segmentation tasks with slight over-segmentation in large tumor cases (see Fig. 10(1,2)). ResUNet++ and SmaAt-Net are unable to capture the tumor region with better edge precision in large tumor segmentation due to minor under-segmentation (see Fig. 10(1)) which is regarded as false negatives. We noticed that multiple tumor sample segmentation was not performed with better edge precision by any comparative model. However, the proposed MANet architecture could capture all the tumor regions compared to the other state-of-the-art methods.

### Feature visualization

**Figure 11:**
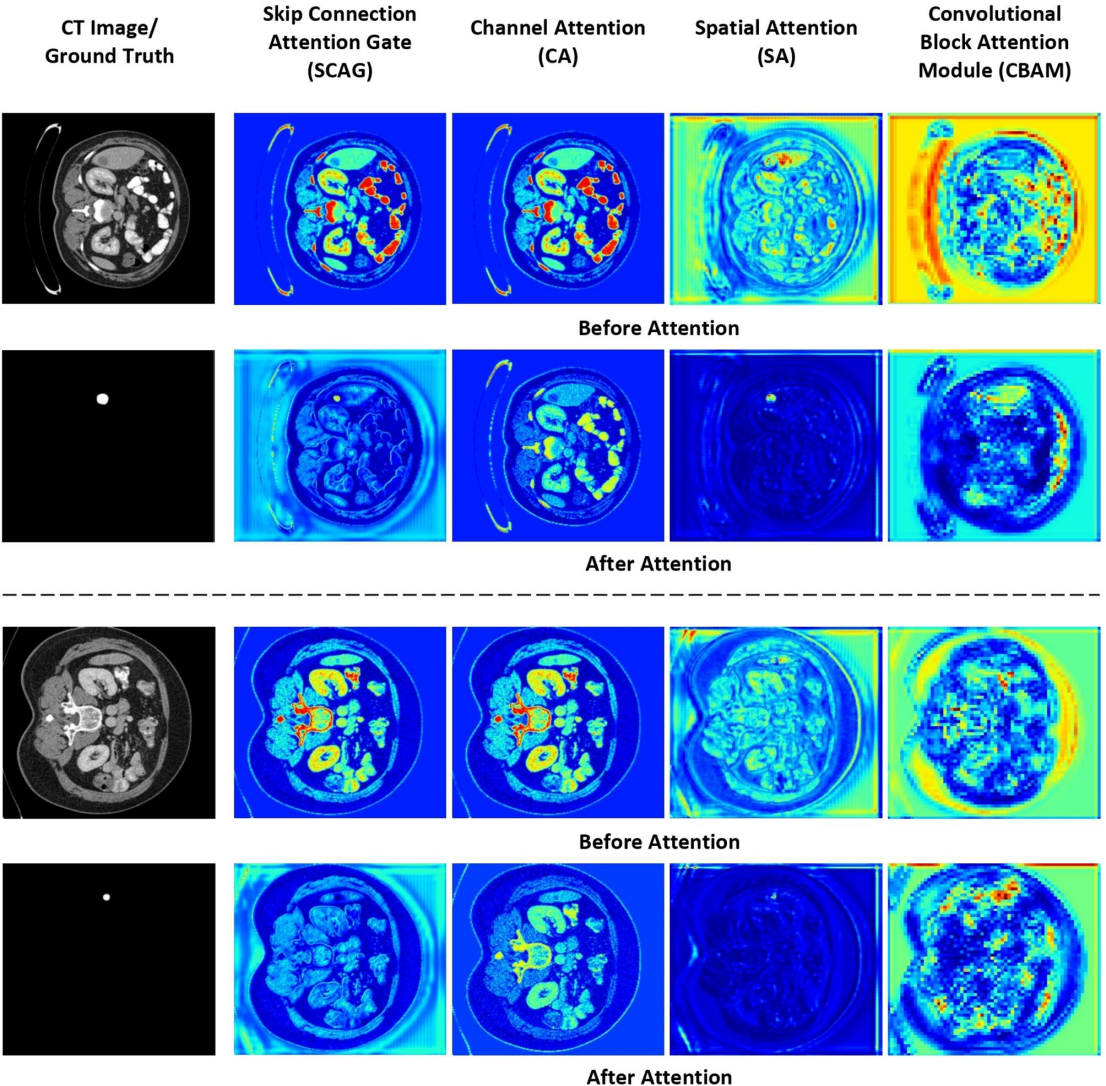
Feature visualization before and after the Skip Connection Attention Gate (SCAG), Channel Attention (CA), Spatial Attention (SA), and Convolutional Block Attention Module (CBAM) used in MANet architecture design.

**Figure 12:**
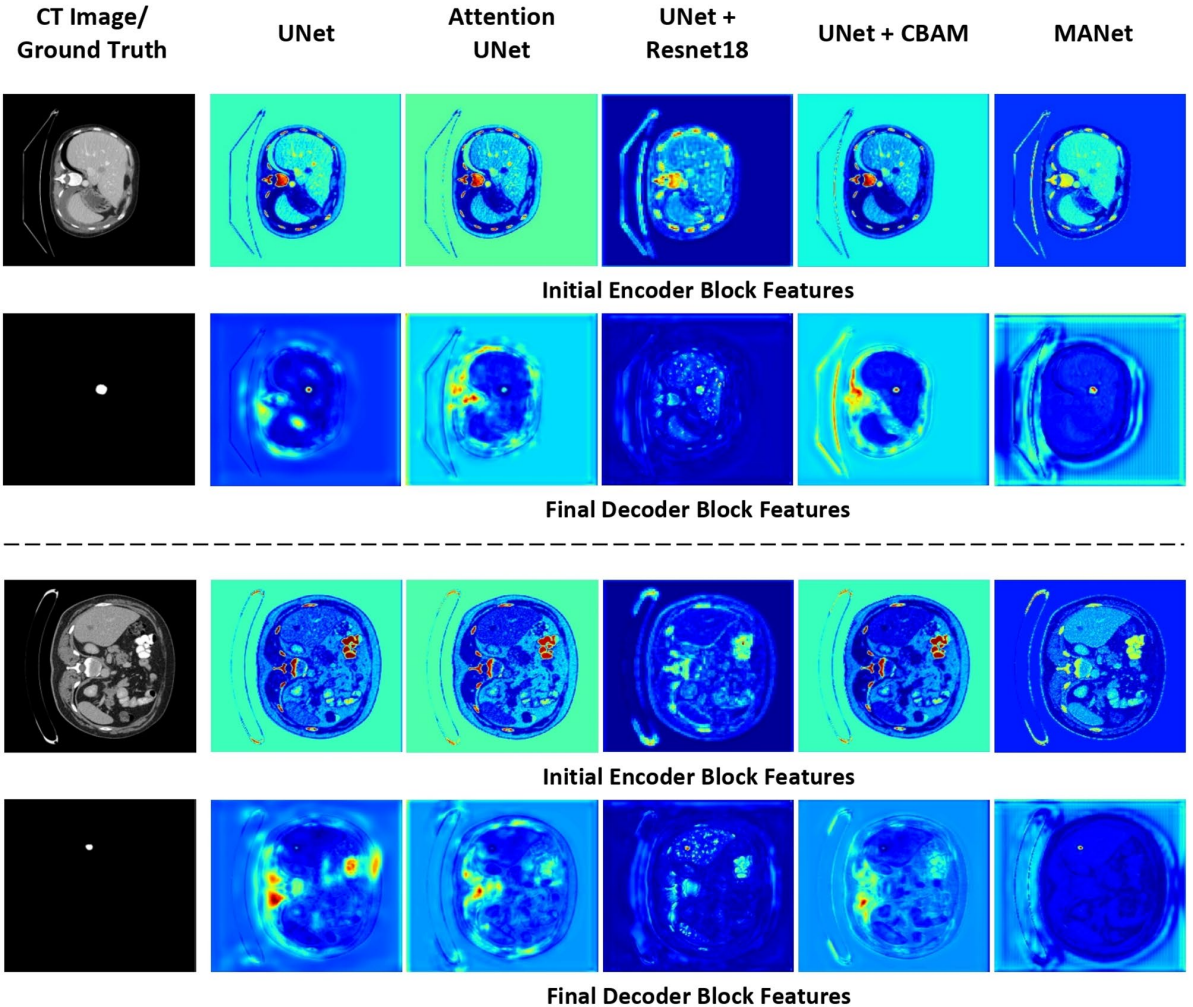
Visualization of corresponding feature maps of comparison networks.

To evaluate the effectiveness of attention mechanisms implemented in the proposed MANet architecture, before and after the attention mechanism features visualized in Fig. 11. The corresponding feature maps of the initial encoder block and the final decoder block of comparison networks are visualized in Fig. 12. All the feature maps are generated by mapping features in between the maximum and minimum value of the features.

The proposed MANet architecture is designed with channel-wise attention mechanisms and spatial-wise attention mechanisms. Channel attention is implemented in the encoder path to highlight important feature maps while suppressing irrelevant feature maps to the liver tumor segmentation task. Channel attention is minimized by the weights in irrelevant regions of the segmentation as visualized in Fig. 11. The convolutional block attention module (CBAM) is initiated focusing on the region of interest (ROI) at the deepest level of the network. The network continued to focus on ROI in the decoder path with the implementation of spatial attention. As visualized in Fig. 11, spatial attention is absolutely suppressed or ignored irrelevant features in the spatial dimension. The skip connection attention gate (SCAG) designed with spatial attention, creates the focus on the ROI of the target to extract important features from the encoder path to concatenate with the deep features in the decoder path.

To illustrate fair evaluation with the comparison models, corresponding feature maps of the initial encoder block and the final decoder block are visualized (see Fig. 12). The proposed MANet demonstrated comparatively better focus on the ROI of the target while suppressing irrelevant regions of the features. The effectiveness of the channel attention mechanism in the encoder path can be seen in the initial encoder block features compared with comparison models. The superiority in focusing on the ROI of the target is illustrated by the final decoder block features. The proposed MANet architecture demonstrated a comparatively better interpretation for liver tumor segmentation.

### Ablation study

**Table 5:** Comparison of ablation study.

No	Method	Dice score	ASSD	Jaccard Index (IoU)	VOE	Accuracy	Sensitivity (Recall)	Specificity
1	UNet	0.7522 ± 0.178	1.4342 ± 1.320	0.6310 ± 0.190	0.3606 ± 0.190	0.9928 ± 0.006	0.8425 ± 0.204	0.9956 ± 0.003
2	UNet + RB	0.7533 ± 0.182	1.5172 ± 1.395	0.6359 ± 0.192	0.3640 ± 0.192	0.9925 ± 0.006	0.8512 ± 0.202	0.9951 ± 0.004
3	UNet + RB + SCAG	0.7532 ± 0.195	1.4247 ± 1.298	0.6353 ± 0.202	0.3646 ± 0.202	0.9927 ± 0.006	0.8329 ± 0.224	0.9956 ± 0.003
4	UNet + RB + CA	0.8010 ± 0.155	1.0137 ± 1.000	0.6901 ± 0.177	0.3027 ± 0.177	0.9940 ± **0.004**	0.8708 ± **0.173**	0.9965 ± **0.002**
5	UNet + RB + SA	0.7550 ± 0.201	1.1610 ± 1.069	0.6389 ± 0.205	0.3610 ± 0.205	0.9929 ± 0.006	0.8292 ± 0.233	0.9958 ± 0.003
6	UNet + RB + CBAM	0.8006 ± 0.157	0.8842 ± 0.814	0.6897 ± 0.178	0.3038 ± 0.178	0.9938 ± **0.004**	0.8712 ± 0.180	0.9962 ± 0.003
7	UNet + SCAG + CA + SA + CBAM	0.8056 ± 0.153	0.8376 ± 0.733	0.6992 ± 0.174	0.3007 ± 0.174	0.9941 ± **0.004**	0.8715 ± 0.177	0.9967 ± 0.003
**8**	**MANet: UNet + RB + SCAG + CA + SA + CBAM**	**0.8145** ±** 0.150**	**0.7084** ±** 0.701**	**0.7084** ± **0.171**	**0.2915** ± **0.171**	**0.9947** ± **0.004**	**0.8723** ± **0.173**	**0.9970** ± **0.002**

The result from MANet and the best values are in bold.

To evaluate the effectiveness of the proposed MANet, we performed an ablation study in 8 steps (Table 5). The UNet is the baseline for the proposed architecture. The convolutional layers in the encoder of UNet were replaced by residual blocks (RB) for the second experiment (UNet+RB). The effectiveness of each attention mechanism is evaluated by integrating it into UNet+RB (i.e., No.3, 4,5, and 6) by considering UNet+RB as the backbone of the proposed network. The effectiveness of the integration of all the attention mechanisms was evaluated with the base model UNet (i.e., No.7) which further demonstrated the impact of the residual structure on the optimality of the proposed MANet architecture.

The ablation experiments result in Table 5 proves that the developments in architecture are beneficial to improve the performance of the network. The residual structure has demonstrated slight improvements in experiment No.2. However, it has further demonstrated the importance of residual blocks in experiment No.7, since the integration of all the attention mechanisms to base model UNet could not outperform the proposed MANet architecture with residual structure. The channel attention (CA) among the attention mechanisms demonstrated a significant performance boost to the proposed architecture. It is further evidenced in experiment No.6 which is the implementation of the backbone with convolutional block attention module (CBAM), that is the combination of channel attention (CA) and spatial attention (SA). It can be seen that combination of UNet, Residual block, and attention mechanism demonstrate better feature extraction compared to the integration of a single mechanism to base model UNet. We can conclude that the fusion of deep learning techniques in MANet could gain a performance boost to liver tumor segmentation.

### Computational cost and future direction

**Table 6:** Comparison of the effectiveness of comparison networks.

Network	Computational complexity (MACs(G))	Total training parameters (M)	Inference time (ms)
UNet	**94.45**	13.37	41.60
Attention UNet	97.07	**6.34**	38.80
UNet + Resnet18	119.14	17.85	**36.20**
UNet + CBAM	166.80	8.39	82.60
MANet (Proposed model)	132.37	7.83	81.80

The best values in bold.

We have calculated computation complexity, total parameter counts, and the inference time per slice to compare the proposed model with the baseline models (Table 6). The UNet architecture takes 41.6 ms inference time to forward propagate one slice and that has the minimum model complexity. However, it requires a comparatively greater amount of memory due to the higher parameter count which is roughly similar to double of parameters in the proposed model. Attention UNet contains the lowest amount of parameter count of 6.34 M with comparatively lower inference time, the model is slightly higher in computational complexity compared to UNet. UNet+Resnet18 architecture has the best inference time (i.e., 36.2 ms) while having the highest parameter count of 17.84 M. UNet+CBAM model has the highest computational complexity (i.e., 166.8 GMac) while the proposed MANet is almost closer to it. Computational complexity in the proposed model is increased due to applying channel attention, spatial attention, and CBAM in the network in all stages. We can note that the computational complexity and parameter count of the proposed architecture could be slightly reduced compared to UNet+CBAM by utilizing channel attention and spatial attention separately in the encoder and decoder path respectively, instead of applying CBAM to all the stages of the network. Overall, the proposed architecture manifested comparatively better segmentation performance by utilizing roughly half of the parameter count of the base model of UNet.

In the future, we aim to evaluate the model with more datasets to validate the generalizability of model. Moreover, we conduct experiments to evaluate the model’s capability in segmentation of the liver and other organs with tumors (i.e., kidney, kidney tumors) with different medical imaging modalities like MRI, PET, and US. Our goal is to develop this architecture utilizing the latest deep learning state-of-the-art techniques to minimize computational complexity while improving the segmentation performance with a higher level of stability.

## Conclusion

In this study we have presented a multi-attention network (MANet) for automatic liver tumor segmentation based on UNet, which can be beneficial to radiologist to perform tumor diagnosis and tumor burden analysis for patient treatment planning. The proposed architecture is strengthened with attention mechanisms and residual learning. In particular, we use channel attention to recalibrate features in channels while spatial attentions to capture localization features of the tumors. Moreover, skip connection attention gates implemented to extract highly relevant low level semantic features to propagate high level semantic information. In addition, residual learning employed in encoder to minimize the degradation to improve the gradient flow. We have evaluated the effectiveness of the proposed design on the LiTS17 dataset and 3DIRCADb dataset by conducting slice-based segmentation and volume-based segmentation experiments, that further demonstrated superiority of our network compared to baseline methods. Moreover, we have conducted empirical study to compare the quantitative and qualitative study to evaluate the performance, which could be further validated the effectiveness and robustness of the multilevel attention strategies implemented in the proposed design. Moreover, the effectiveness of the proposed architecture is further evaluated in the ablation study. However, we should note that there was a significant performance gap in between slice-based segmentation and volume-based segmentation. This should be the challenges due to high variability in data sources in terms of the shape of liver tumors and intensity variations. Therefore, there are important issues to be addressed to generalize the model to perform volume-based segmentation in real clinical environment.

## Data Availability

The datasets generated and/or analysed during the current study are available in the MICCAI 2017 Liver Tumor Segmentation Challenge (LiTS17) repository^20^, https://competitions.codalab.org/competitions/17094 and 3D-IRCADb-01 repository^21^, https://www.ircad.fr/research/data-sets/liver-segmentation-3d-ircadb-01/.
